# Supplementary figures and images for: Transcriptomic Analysis of Resistant and Susceptible Responses in a New Model Root-Knot Nematode Infection System Using Solanum torvum and Meloidogyne arenaria
Source: Front Plant Sci. 2021 May 25;12:680151. doi: 10.3389/fpls.2021.680151 (PMC8194700; doi:10.3389/fpls.2021.680151)

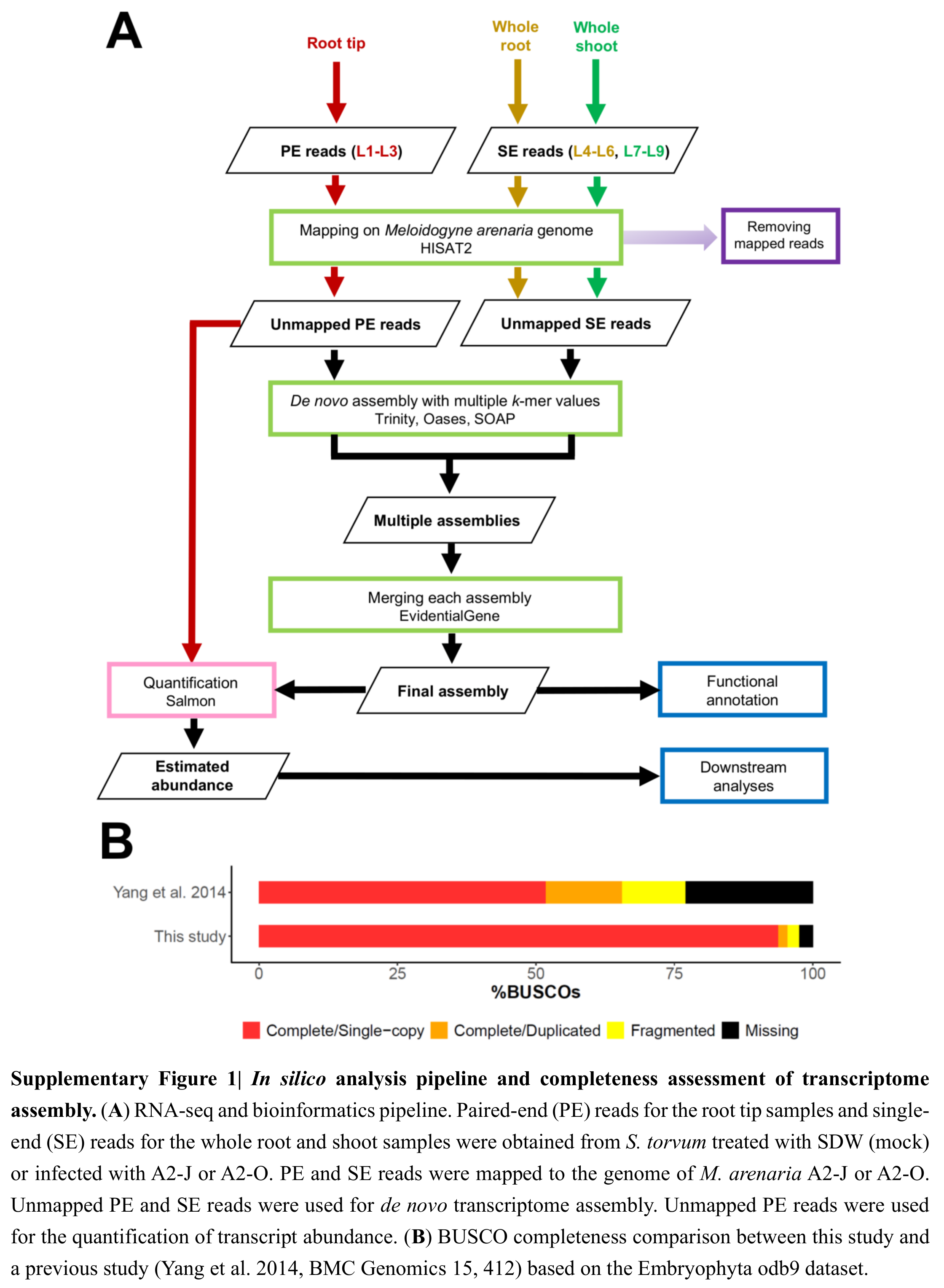

Supplement: Supplementary file 1 [file Image_1.TIF]

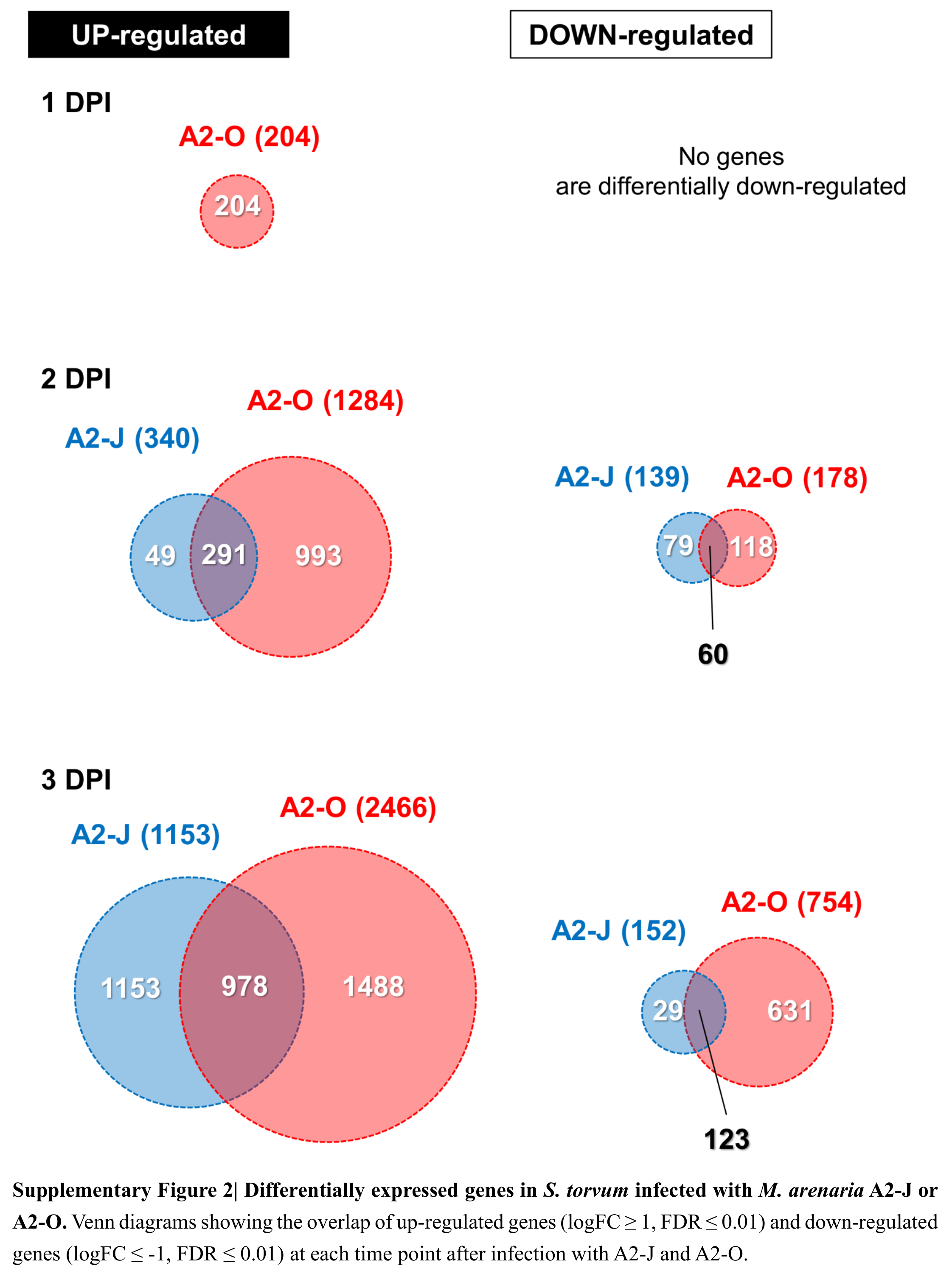

Supplement: Supplementary file 2 [file Image_2.TIF]

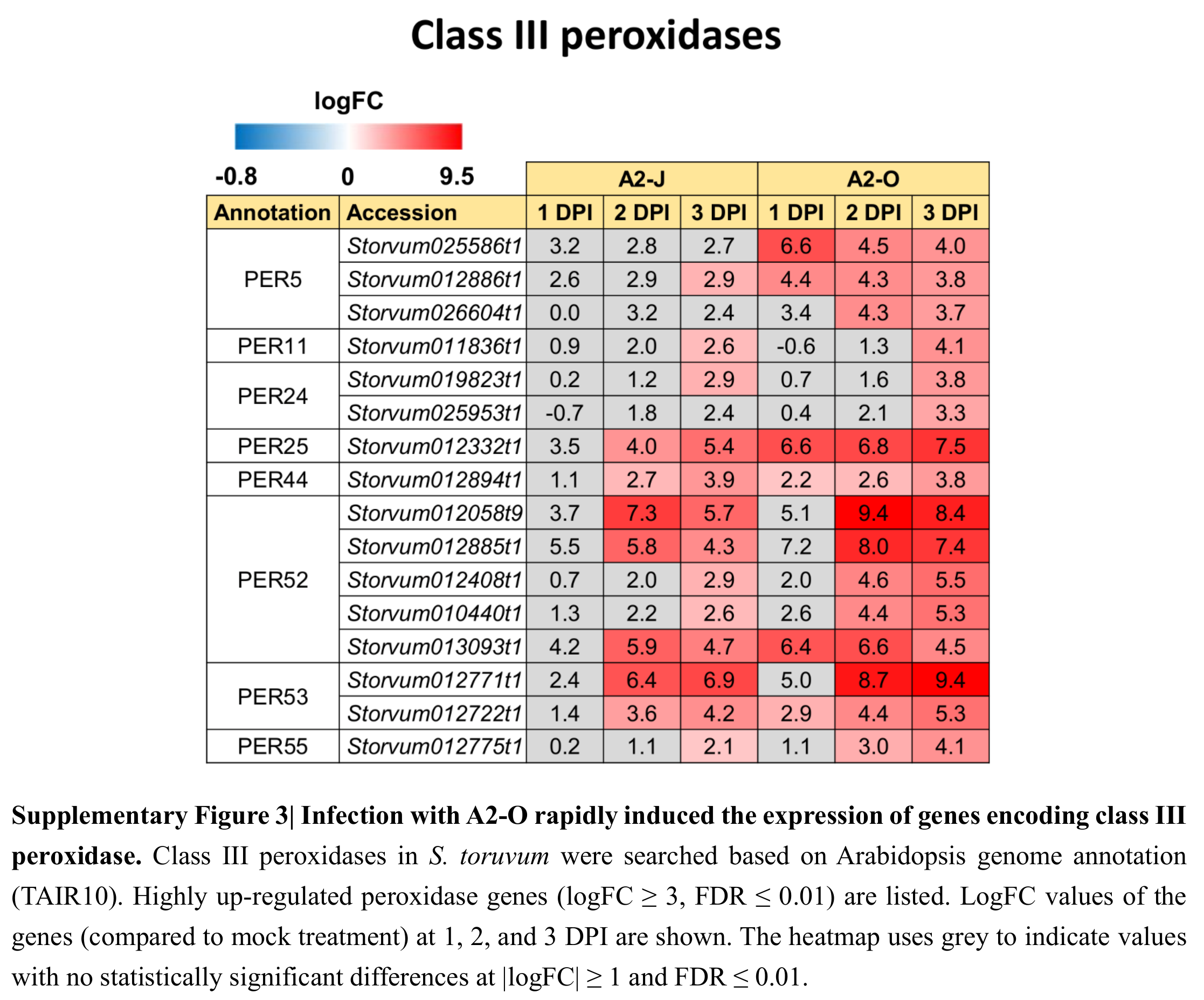

Supplement: Supplementary file 3 [file Image_3.TIF]

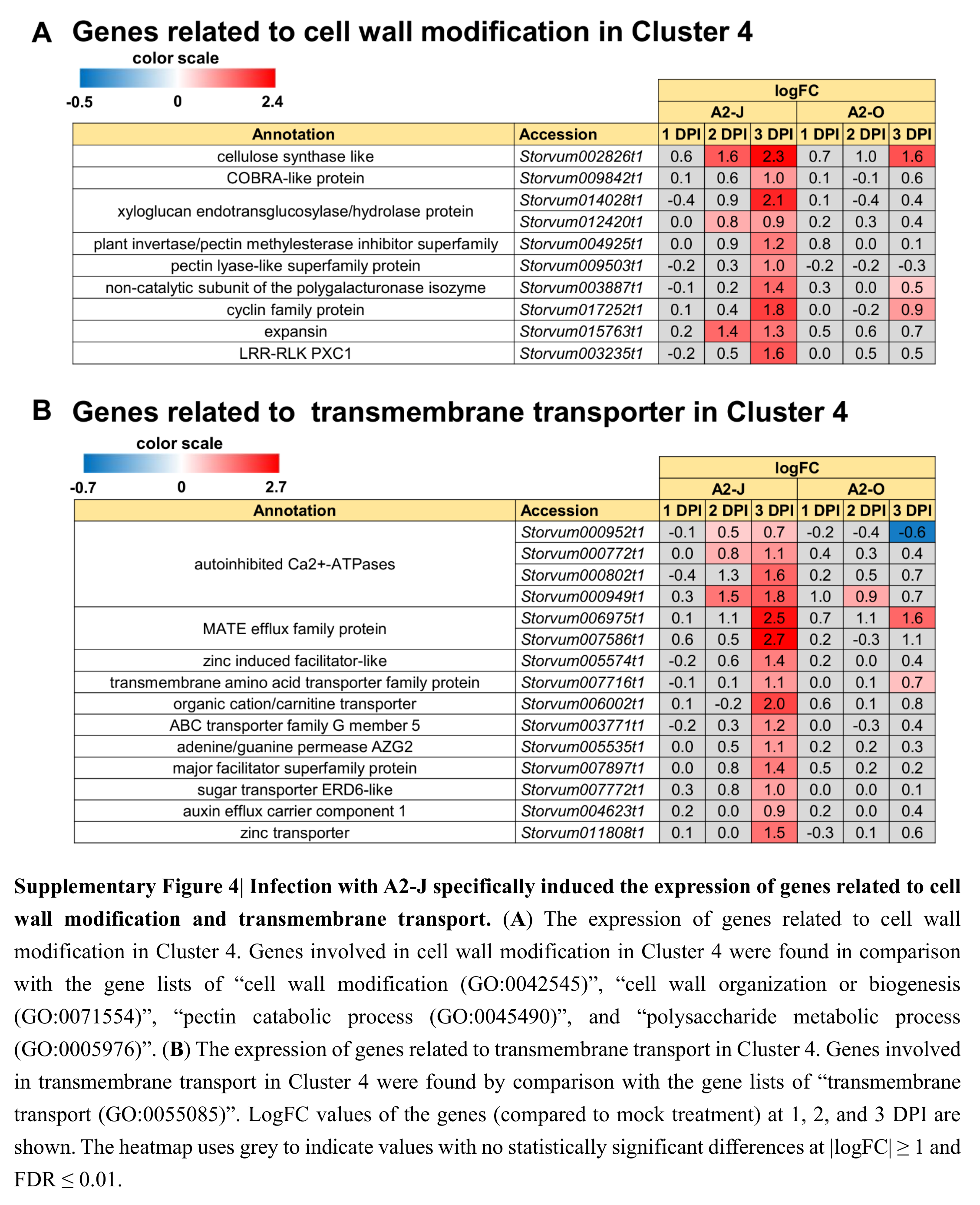

Supplement: Supplementary file 4 [file Image_4.TIF]

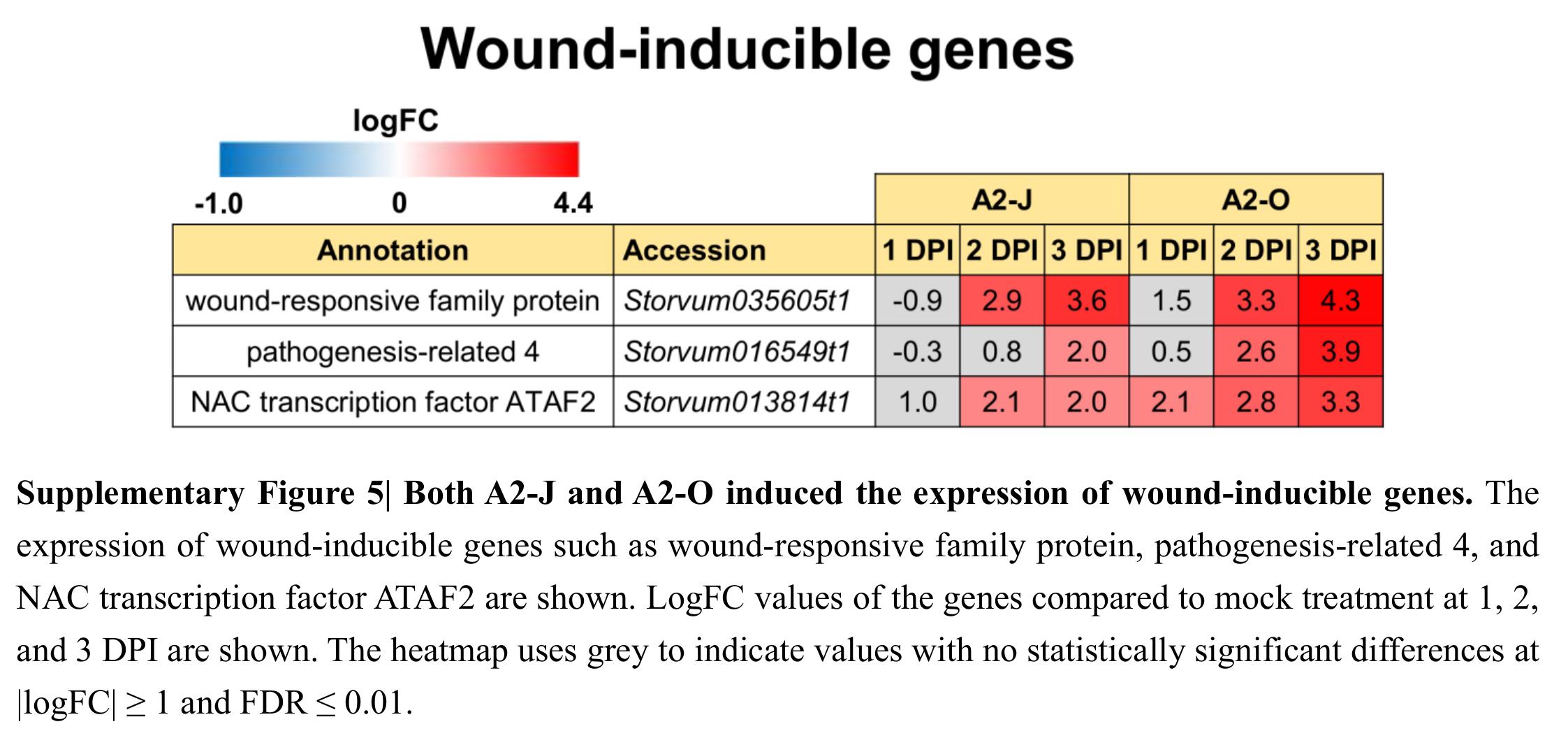

Supplement: Supplementary file 5 [file Image_5.TIF]

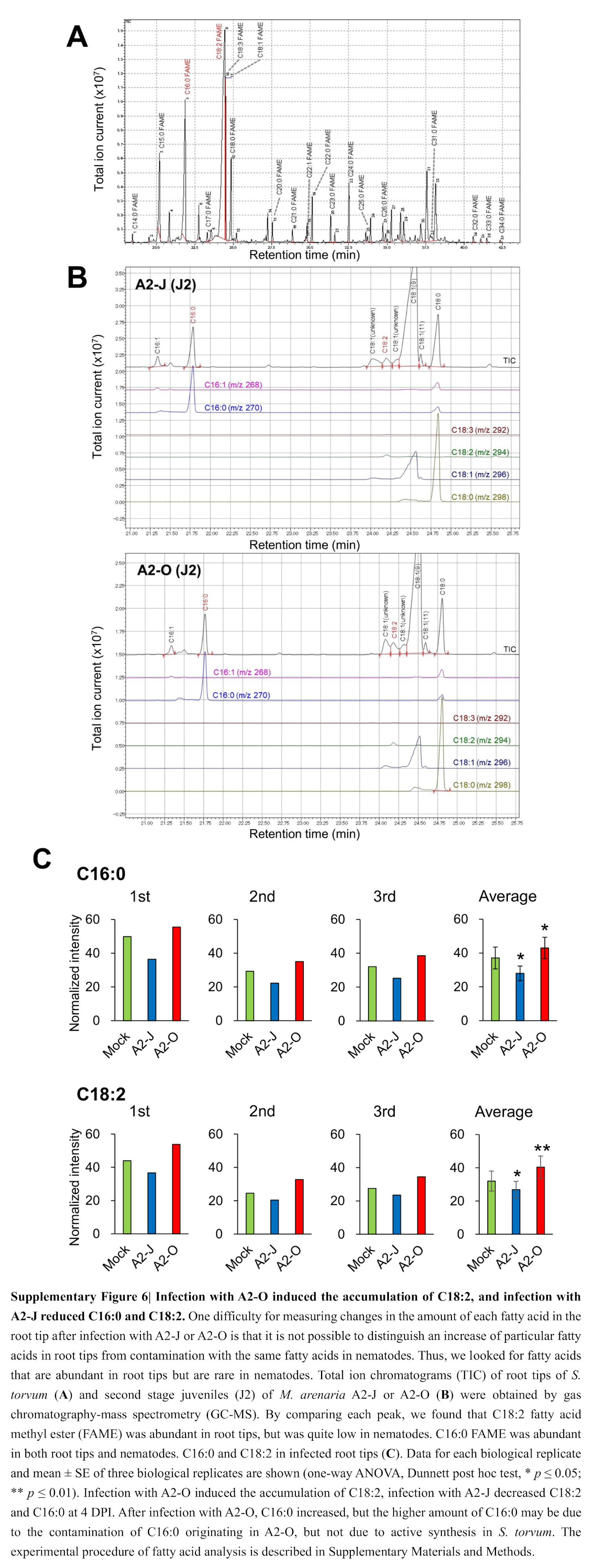

Supplement: Supplementary file 6 [file Image_6.TIF]
